# Inhibition of Connexin43 Hemichannels Impairs Spatial Short-Term Memory without Affecting Spatial Working Memory

**DOI:** 10.3389/fncel.2016.00288

**Published:** 2016-12-20

**Authors:** Laura Walrave, Mathieu Vinken, Giulia Albertini, Dimitri De Bundel, Luc Leybaert, Ilse J. Smolders

**Affiliations:** ^1^Department of Pharmaceutical Chemistry, Drug Analysis and Drug Information, Center for Neurosciences, Vrije Universiteit BrusselBrussels, Belgium; ^2^Department of In Vitro Toxicology and Dermato-Cosmetology, Vrije Universiteit BrusselBrussels, Belgium; ^3^Physiology Group, Department of Basic Medical Sciences, Ghent UniversityGhent, Belgium

**Keywords:** connexin43, Y maze, TAT-Gap19, working memory, short-term memory

## Abstract

Astrocytes are active players in higher brain function as they can release gliotransmitters, which are essential for synaptic plasticity. Various mechanisms have been proposed for gliotransmission, including vesicular mechanisms as well as non-vesicular ones, for example by passive diffusion via connexin hemichannels (HCs). We here investigated whether interfering with connexin43 (Cx43) HCs influenced hippocampal spatial memory. We made use of the peptide Gap19 that blocks HCs but not gap junction channels and is specific for Cx43. To this end, we microinfused transactivator of transcription linked Gap19 (TAT-Gap19) into the brain ventricle of male NMRI mice and assessed spatial memory in a Y maze. We found that the *in vivo* blockade of Cx43 HCs did not affect the locomotor activity or spatial working memory in a spontaneous alternation Y maze task. Cx43 blockade did however significantly impair the spatial short-term memory in a delayed spontaneous alternation Y maze task. These results indicate that Cx43 HCs play a role in spatial short-term memory.

## Introduction

Over the few last years, evidence has accumulated pointing to a key role for connexin43 (Cx43) signaling in neurophysiology (Cheung et al., 2014). Cx43 forms gap junctions (GJs) and hemichannels (HCs), which mediate intercellular and extracellular communication, respectively (Chandrasekhar and Bera, 2012). In the central nervous system, Cx43 is expressed in developing neurons, activated microglia, pericytes and endothelial cells of blood vessels, but it is predominantly and abundantly found in astrocytes (Naus et al., 1991; Nagy and Rash, 2000; Contreras et al., 2004; Chew et al., 2010; Winkler et al., 2011).

While astrocytic Cx43 GJs connect two adjacent astrocytes and are normally open under physiological circumstances, astrocytic Cx43 HCs are located between the cytosol of a single astrocyte and its extracellular environment and have long been assumed to be inactive in basal conditions due to their low open probability at resting membrane potential and baseline ionic concentrations (Contreras et al., 2003; Giaume et al., 2013; De Bock et al., 2014). Accordingly, astrocytic HCs seem predominantly active in pathological conditions, e.g., ischemia and inflammation, where they are proposed to be involved in damage-associated adenosine triphosphate (ATP) release, disturbed transmembrane ion fluxes and loss of essential metabolites (Giaume et al., 2013; De Bock et al., 2014; Wei et al., 2014; Montero and Orellana, 2015).

Work by Torres et al. (2012) on acute hippocampal slices however demonstrated that lowering the extracellular calcium ion (Ca^2+^) concentration to levels that can reasonably be attained during neuronal burst activity, triggers Cx43-dependent ATP release that subsequently influences the activity of inhibitory neurons. This work suggested that astrocytic Cx43 HC opening, gliotransmitter release and modulation of synaptic signaling might be operational under physiological conditions. In line with this idea, it has been shown that astroglial Cx43 HCs can open under resting conditions and modulate basal synaptic transmission through ATP (Chever et al., 2014a) and glutamate (Chever et al., 2014b) signaling and that deletion of astroglial Cxs alters synaptic transmission and plasticity (Pannasch and Rouach, 2013). Additionally, Stehberg et al. (2012) recently demonstrated that *in vivo* modulation of Cx43 HCs alters fear memory. For this purpose, they applied the transactivator of transcription linked L2 peptide (TAT-L2), a selective Cx43 HC inhibitor, in the basolateral amygdalae, which blocked fear memory consolidation. Fear learning capacity was recovered after co-infusing a cocktail of gliotransmitters (glutamate, D-serine, glutamine, ATP, lactate and glycine). These experiments substantiate a critical role for *in vivo* gliotransmission mediated by Cx43 HCs in fear memory. Indeed, it is known that changes in neuronal plasticity, a substrate for memory (e.g., long-term potentiation), can be modulated by astrocytic gliotransmitter release (Ben Achour and Pascual, 2010; Moraga-Amaro et al., 2014).

**Figure 1:**
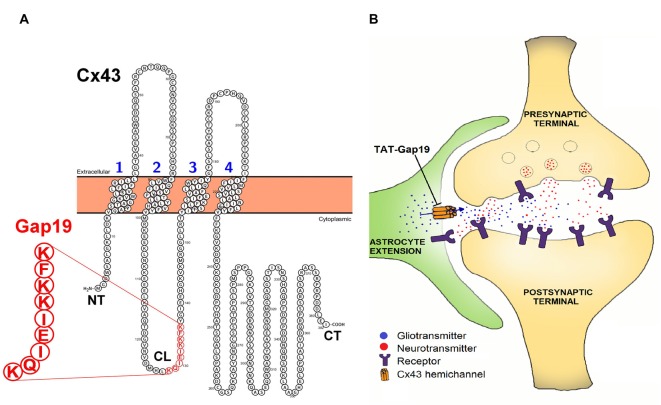
**(A)** Position of the Gap19 sequence (red) in the cytoplasmic loop (CL) domain of connexin43 (Cx43). Gap19 interacts with the last nine amino acids of the carboxyl terminus (CT) tail, and thereby inhibits Cx43 hemichannel (HC) opening (Illustration generated with the Protter tool (Omasits et al., 2014)) and **(B)** the tripartite synapse (presynaptic neuron—postsynaptic neuron—astrocyte) in which astrocytic Cx43 hemichannels (HCs) may contribute by releasing neuroactive substances (gliotransmitters), that act as a feedforward signal generated from distant neurons/glial cells or that act in feedback to the synapse upon astrocyte activation by neurotransmitters. HC opening is inhibited by the selective Cx43 HC inhibitor, transactivator of transcription (TAT)-Gap19.

The aim of our present work is to determine whether Cx43 HCs play a role in spatial memory. To this end, we made use of Gap19, a synthetic nonapeptide derived from the intracellular L2 domain of Cx43 that specifically inhibits intramolecular cytoplasmic loop (CL)/C-terminal tail interactions, which are essential for Cx43 HCs to open (Wang et al., 2013b; Figure 1). The basis for the HC specificity relates to the fact that CL/C-terminal tail interactions distinctly affect GJs and HCs, with GJs being closed by these interactions while HCs need these interactions to become available to open with electrical or chemical triggers (Iyyathurai et al., 2013; Wang et al., 2013b). By binding to the C-terminal tail, Gap19 prevents CL/C-terminal tail interactions and thereby keeps Cx43 HCs closed and refractive to potential opening triggers (Wang et al., 2013b). The specificity of Gap19 for Cx43 relates to the fact that the target of Gap19 (i.e., the C-terminal tail) is intracellularly located and the intracellular domains of the Cx proteins are the least conserved regions in contrast to the extracellular domains which are targeted by other Cx43 mimetic peptides (e.g., Gap26 and Gap27; Wang et al., 2013a).

Astrocytes can be excited by an elevation of the intracellular Ca^2+^ concentration ([Ca^2+^]_i_)_,_ which also triggers opening of HCs. Indeed, [Ca^2+^]_i_ elevation up to 500 nM triggers the opening of Cx43 HCs, while higher concentrations close the channels again (De Vuyst et al., 2009). This high [Ca^2+^]_i_ closure of HCs may act as a brake to prevent excessive, pathological HC opening, suggesting these channels may have physiological roles. Interestingly, Gap19 not only inhibits voltage-gated Cx43 HC opening but also [Ca^2+^]_i_-elevation induced Cx43 HC opening (Wang et al., 2013a).

In this study, we microinfused transactivator of transcription (TAT)-Gap19 in the murine brain ventricle and tested its effects on spatial working memory and spatial short-term memory in a Y maze. Working memory refers to memory as it is used to plan and carry out behavior (Cowan, 2008). It is defined as the maintenance and controlled manipulation of a limited amount of information before recall (Aben et al., 2012). Short-term memory reflects faculties of the mind that can hold a limited amount of information (chunck capacity limits) in a very accessible temporarily state (duration limits; Cowan, 2008). Our results demonstrate that inhibition of Cx43 HCs with TAT-Gap19 impaired spatial short-term memory without affecting locomotor activity or spatial working memory.

## Materials and Methods

### Animals

All experiments were performed on male NMRI mice (Charles River Laboratories, Chatillon-sur-Chalaronne, France) weighing 20–25 g at the time of surgery (~4 weeks old). The animals were housed in a temperature/humidity regulated environment with a 10/14 h light/dark cycle and received food pellets and water *ad libitum*. The mice habituated 1 week to the animal house and 1 day to the experimental room before being used in experiments. All procedures were carried out in accordance with the National Rules on Animal Experiments and were approved by the Ethical Committee for Animal Experiments of the Faculty of Medicine and Pharmacy of the Vrije Universiteit Brussel, Brussels, Belgium. To the best of our abilities, results were reported in accordance with the ARRIVE guidelines (Kilkenny et al., 2010).

### Surgical Implantation of an Intracerebroventricular (i.c.v.) Guide Cannula

NMRI mice were anesthetized in an induction chamber with 4% isoflurane (Iso-vet^®^, 1000 mg/g isoflurane, Dechra Veterinary Products, Bladel, Netherlands). After induction, anesthesia was maintained during the entire duration of the surgery by 2.5%–3% isoflurane via a facemask. Ketoprofen (3 mg/kg, Ketofen^®^, 10 mg/mL ketoprofen, Merial, Toulouse, France) was administered subcutaneously at the beginning of the surgical procedure to prevent post-operative pain and inflammation. A guide cannula (3 mm, 26 GA, Bilaney Consultants, Düsseldorf, Germany) was implanted stereotactically in the left murine brain ventricle, using the following coordinates relative to bregma: +1 mm medial-lateral, −0.34 mm anterior-posterior and −2.20 mm ventral-dorsal.

After surgery, the mice received 1 mL saline (0.9% NaCl, Baxter, Lessines, Belgium) intraperitoneally and were placed in front of an infrared lamp for 15 min to raise the core body temperature. They were single-housed to prevent cannulated mice from chewing on each other’s cannulas and were allowed to recover for 7 days. A stylus (Bilaney Consultants) was placed inside the guide cannula to prevent clogging.

**Figure 2:**
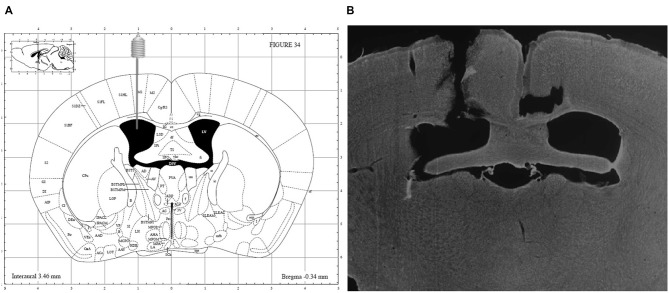
**(A)** Schematic overview of i.c.v. guide implantation with following coordinates relative to bregma: +1 mm medial-lateral, −0.34 mm anterior-posterior and −2.20 mm ventral-dorsal (Paxinos and Franklin, 2004) and **(B)** representative image of a DAPI counterstained coronal brain section showing accuracy of guide implantation in the left ventricle.

At the end of the experiments, the mice were sacrificed using an overdose of sodium pentobarbital (200 mg/kg, Nembutal^®^, Ceva Santé Animale, Libourne, France) and accuracy of guide implantation was verified postmortem and compared against a mouse brain atlas (Paxinos and Franklin, 2004). A representative image is depicted in Figure 2.

### Drugs Used and Their Administration

We used Gap19 coupled to the TAT membrane translocation motif in order to increase its cell membrane permeability, as the C-terminal tail is intracellularly located (Abudara et al., 2014). TAT-Gap19I130A, an I130A-modified Gap19 analog, was chosen as a negative control peptide since amino acid I130 is involved in the formation of hydrogen bonds and thereby important for Gap19 activity (Wang et al., 2013b). Hence, TAT-Gap19I130A exerts no inhibitory effects on Cx43 HCs (Wang et al., 2013b). Scopolamine, a cholinergic M1 (muscarinic) receptor antagonist, which is often used to generate deficits of performance in the Y maze and other memory tests, was used as a positive control (Kwon et al., 2010; Ohba et al., 2015). Biotin-labeled TAT-Gap19 (biotin-TAT-Gap19) was used to determine peptide distribution following i.c.v. injection.

TAT-Gap19 (MW 2703.28 Da), biotin-TAT-Gap19 (MW 2929.61 Da) and the inactive mutant version TAT-Gap19I130A were synthesized by Pepnome Inc. (Hong Kong, China, >95% purity) and dissolved in phosphate buffered saline (PBS; Sigma-Aldrich, Steinheim, Germany), at a final concentration of 1 mM. Scopolamine hydrobromide trihydrate was supplied by Sigma-Aldrich and dissolved in PBS to yield a final concentration of 33 mM. On the day of the experiment, the stylus was removed from the guide cannula and a 33 GA injection cannula (Bilaney Consultants) was inserted through the guide cannula, extending 1 mm beyond its tip in the ventricle. Compounds were i.c.v. infused via the injection cannula for 2 min at a flow rate of 0.5 μL/min (total 1 μL), driven by a microinjection pump (CMA 400 Syringe pump, CMA/Microdialysis AB), 60 min prior to testing in the Y maze. Following drug infusion, injection cannulas were left in place for 1 min to allow drug diffusion away from the cannula tip. Doses were based on literature (e.g., Stehberg et al., 2012; Abbasi et al., 2013; Tabari et al., 2016). For TAT-Gap19 and TAT-Gap19I130A, mice received 1 nmol/μL or 2.7 μg/μL i.c.v. For scopolamine hydrobromide trihydrate, mice received 33 nmol/μL or 10 μg/μL scopolamine i.c.v.

### Validated Paradigms for Testing Spatial Working and Short-Term Memory in a Y Maze

Behavioral testing was performed in a Y maze with three identical, opaque arms at 120° angle from each other, as previously described (De Bundel et al., 2011). The arms were 35 cm long, 5 cm wide and 8 cm high, allowing the mice to see distal spatial cues. The Y maze was swabbed with 70% ethanol between each test to eliminate odors. Solutions were prepared and syringes were filled by one experimenter. Another experimenter encoded the syringes with a number and animals were randomized between these numbers. At the time of data processing and data analyses, the experimenter was blinded to the treatment group.

The continuous spontaneous alternation testing is based on the natural tendency of rodents to explore a novel environment. The mice were placed in the Y maze, facing the wall of one randomly chosen arm and were allowed to freely explore the three arms of the Y maze for 8 min. Typically, mice explore the least recently visited arm and tend to alternate between the three arms. For efficient alternation, mice rely on their working memory (Wall and Messier, 2002). Every arm entry was recorded manually in order to calculate the percentage of alternation. An entry occurred when all four limbs were within the arm. The total number of arm entries was used as a measure for locomotor activity, while the spontaneous alternation percentage (SAP) was used as a measure of spatial working memory. To calculate the SAP, the total number of alternations (i.e., every time a mouse explored the three arms consecutively) was divided by the total possible alternations (i.e., the number of arm entries minus 2) and multiplied by 100. Immediate reentries were discounted.

In the delayed spontaneous alternation testing, one of the three arms was closed and the mice were allowed to explore the other two arms for 15 min (training phase). Next, the mice were transferred to their holding cage for 5 min. Subsequently, they were placed into the start arm and were allowed to explore all three arms of the maze for 5 min (test phase). During these 5 min, the time spent in each arm was recorded manually and the time spent in the novel arm (i.e., previously closed during the training phase) was used as a measure for spatial short-term memory.

### Diffusion of Biotin-Labeled TAT-Gap19 into the Mouse Brain

Peptide diffusion following i.c.v. delivery was determined by injecting 1 μL of 1 mM biotin-TAT-Gap19 (1 nmol/μL or 2.9 μg/μL) or 1 μL PBS (control). Immunohistochemistry was performed 60 min after the injection, in accordance with the *in vivo* protocol.

Briefly, anesthetized mice (200 mg/kg sodium pentobarbital i.p., Nembutal^®^) were intracardially perfused for 2 min with 0.9% NaCl and 3 min with 4% paraformaldehyde (Sigma-Aldrich) in PBS. Following perfusion, the brains were removed, post-fixed in 4% paraformaldehyde and sliced into 40 μm coronal sections using a vibratome (Leica VT1000S, Leica Biosystems, Diegem, Belgium).

All rinsing steps and incubations were performed in Tris-buffered saline with Triton-X-100 (TBST; 0.01 M Tris, 0.1% Triton-X-100, 0.9% NaCl, pH 7.4; all supplied by Sigma-Aldrich) at room temperature and under gentle agitation, unless stated otherwise. Free-floating sections were washed with TBST (three times 10 min) and blocked with 10% pre-immune goat (PIG) serum (Milllipore, Temecula, CA, USA) in TBST for 45 min. The sections were then incubated with primary polyclonal rabbit anti-GFAP antibody (diluted 1:1000 in 10% PIG in TBST, cat. No. Z0334, Dako, Glostrup, Denmark) overnight at 4°C. The next day, sections were washed with TBST (three times 10 min) and incubated with secondary goat anti-rabbit IgG linked to Alexa Fluor 488 (diluted 1:500 in TBST, cat. No. 111-545-003, Jackson ImmunoReserach Laboratories, Inc., PA, USA) and streptavidin-labeled CY3 (diluted 1:20 in TBST, Cat. No 434315, Invitrogen, Life Technologies, Ghent, Belgium). Following two washing steps with TBST (each 10 min), nuclei were counterstained with DAPI (1 μg/mL in TBST, Cat. No. D3571, Life Technologies, Ghent, Belgium) for 5 min and finally washed with Tris-buffer (0.01 M Tris, pH 7.4; two times 10 min) before mounting on VWR^®^ Superfrost^®^ Plus Micro slides (VWR International, Leuven, Belgium).

Slides were coverslipped with DPX mounting medium (Sigma-Aldrich) and images were acquired with a Zeiss LSM710 NLO TiSa multiphoton confocal microscope (excitation wavelength lasers: 488 nm, 561 nm and 790 nm), using Zeiss Zen2011 software (Carl Zeiss NV-SA, Zaventem, Belgium).

### Data Analysis and Statistics

Statistical analyses were performed using GraphPad Prism 6.01 with α set at 0.05. The results are expressed as means ± standard error of the mean (SEM). All values were normally distributed (D’Agostino and Pearson omnibus normality test) and a two-sided *t*-test with Welch correction was used to denote any differences between experimental groups.

## Results

### TAT-Gap19 Does Not Influence Spatial Working Memory or Locomotor Activity

We first determined whether i.c.v. administered compounds could reliably affect working memory as measured with our Y maze protocol. This validated protocol measures activity/exploratory motivation and spatial working memory, by analyzing the total number of arm entries and the total percentage of correct spontaneous alternations (SAP), respectively. The cholinergic M1 (muscarinic) receptor antagonist was used to generate deficits of performance in the Y maze (de Bruin and Pouzet, 2006; Kwon et al., 2010; Busquet et al., 2012; Ohba et al., 2015).

**Figure 3:**
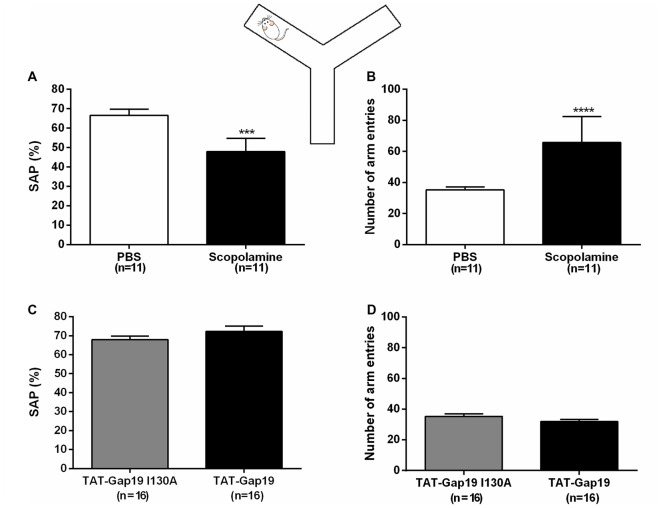
**Spontaneous alternation Y maze protocol.** Effects of i.c.v. administered scopolamine (10 μg/μL) on **(A)** spontaneous alternation and **(B)** number of arm entries in the Y maze, compared to vehicle control (PBS). Effects of i.c.v. administered TAT-Gap19 (2.7 μg/μL) on **(C)** spontaneous alternation and **(B)** number of total arm entries in the Y maze task, compared to the inactive control peptide TAT-Gap19I130A (2.7 μg/μL). While scopolamine induced an impairment in spatial working memory (*p* = 0.0002) and an increase in locomotor activity (*p* < 0.0001), TAT-Gap19 did not alter these parameters in the Y maze. Bars represent **(A,C)** spontaneous alternation percentages (SAP) ± SEM and **(B,D)** total number of arm entries ± SEM.Two-sided *t*-test. ***p < 0.001 and ****p < 0.0001.

All animals showed good ambulatory activity (i.e., above six arm entries). We found that i.c.v. administered scopolamine (10 μg/μL) significantly decreased the amount of correct alternations (SAP) from 66.54% ± 3.25 in control (PBS) to 47.97% ± 2.03 (both *n* = 11; *p* = 0.0002; Figure 3A). Scopolamine also increased the total number of arm entries from 35.27 ± 1.94 in control animals (PBS) to 65.82 ± 5.04 (both *n* = 11; *p* < 0.0001; Figure 3B).

We next tested TAT-Gap19 (2.7 μg/μL or 1 nmol/μL) as well as TAT-Gap19I130A (same concentration), which is composed of an inactive sequence, in the Y maze protocol. No significant differences were observed in SAP between the TAT-Gap19 treated group (72.92% ± 2.75) compared to the TAT-Gap19I130A treated group (67.90% ± 1.93; both *n* = 16, *p* = 0.2028; Figure 3C) and the exploration rates were not affected as the total arm entries in these experimental groups were not significantly different (TAT-Gap19I130A: 35.31 ± 1.73; TAT-Gap19: 31.94 ± 1.45; both *n* = 16, *p* = 0.1459; Figure 3D). These results indicate that TAT-Gap19 does not influence locomotor activity or exploratory motivation and that the peptide does not impair spatial working memory.

### TAT-Gap19 Impairs the Spatial Short-Term Memory

**Figure 4:**
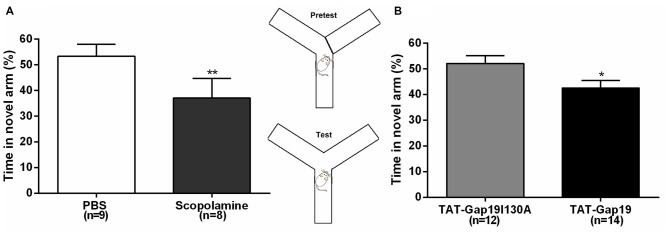
**Delayed spontaneous alternation Y maze protocol.** Effects of i.c.v. **(A)** scopolamine (10 μg/μL) compared to vehicle control (PBS) and **(B)** TAT-Gap19 (2.7 μg/μL) compared to the inactive control peptide TAT-Gap19I130A (2.7 μg/μL) on the time spent in the novel arm. Short-term spatial memory was significantly impaired by scopolamine (*p* = 0.0099) and TAT-Gap19 (*p* = 0.0320). Bars represent percentages (i.e., total time in the novel arm, divided by the total duration of the test (5 min), multiplied by 100) ± SEM. Two-sided *t*-test; *p < 0.05, **p < 0.01.

Next, we used the well-established delayed Y maze protocol to determine effects at the level of short-term spatial memory (Figure 4). I.c.v. administered scopolamine reduced the time spent in the novel arm to 37.08% ± 2.70 (*n* = 8) compared to control (PBS) animals (53.37% ± 4.64; *n* = 9; *p* = 0.0099), validating that i.c.v. administered scopolamine provokes impaired short-term spatial memory in the delayed Y maze protocol (Figure 4A). Next, we tested TAT-Gap19 and found it to significantly reduce the time spent in the novel arm to 42.60% ± 2.94 (*n* = 14), compared to TAT-Gap19I130A (52.14% ± 2.98; *n* = 12, *p* = 0.0320), indicating impaired spatial short-term memory (Figure 4B).

### Biotin-TAT-Gap19 Reaches the Hippocampus Following i.c.v. Injection

**Figure 5:**
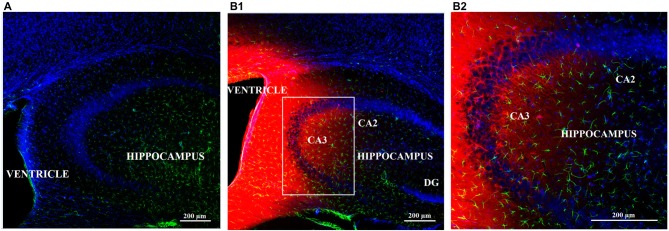
**Diffusion of biotin-TAT-Gap19 into the mouse brain 60 min after injection in the left lateral ventricle.** Representative vibratome coronal sections (40 μm thick) through the left ventricle and hippocampus of mice injected with **(A)** vehicle (PBS) or **(B1,2)** biotin-TAT-Gap19. Sixty minutes after injection, the brain displays clear biotin immunoreactivity (red) around the ventricle and in the hippocampus compared to mice that received PBS only. **(B1)** Area in white box is enlarged in the right box of the panel **(B2)**. Sections were double-labeled with anti-GFAP antibody to visualize astrocytes (green) and nuclei were counterstained with DAPI (blue). (Magnification: **(A,B1)** 10× zoom 0.6 and **(B1)** 10× zoom 1.3).

Following injection of biotin-TAT-Gap19 in the left lateral ventricle, the peptide was detected by using the biotin-streptavidin method. Positive biotin immunoreactivity was observed in the tissue around the left and right lateral ventricle and around the dorsal third ventricle (ventricular zone; data not shown). Additionally, diffuse labeling of biotin-TAT-Gap19 was observed in the hippocampus (Figures 5B1,2), compared to controls only receiving PBS (Figure 5A).

## Discussion

In the present study, we used a spontaneous alternation and delayed spontaneous alternation Y maze test in order to investigate the role of Cx43 HCs in spatial working and spatial short-term memory respectively. The Y maze was chosen because of its simplicity and sensitivity to evaluate spatial memory in mice. The alternation principle is based on the exploratory behavior. Mice prefer to visit the less recently visited arm, implicating they need to remember the last arm they visited (Sharma et al., 2010).

The Cx43 mimetic peptide TAT-Gap19 is one of the sole currently available tools to discriminate Cx43 GJs and HCs. The applied dose of TAT-Gap19 is comparable to the dose of TAT-L2 used by Stehberg and team (Stehberg et al., 2012). In that study, TAT-L2 was micro-infused in both basolateral amygdalae (total 0.5 nmol) to study the role of Cx43 HCs in fear memory, which is mainly amygdala-dependent. Due to diffusion and dilution effects from the ventricle to other brain regions (e.g., hippocampi), we administered 1 nmol TAT-Gap19. When distributed in an estimated 35–40 μL cerebrospinal fluid volume (Pardridge, 1991; Johanson et al., 2008), this gives an expected 29–25 μM concentration (i.e., four times the half-maximal concentration (i.e., 7 μM, Abudara et al., 2014)).

Both experimental protocols (spontaneous and delayed spontaneous alternation) were validated with i.c.v. administered vehicle (PBS) and scopolamine (de Bruin and Pouzet, 2006; Kwon et al., 2010; Busquet et al., 2012; Ohba et al., 2015). Scopolamine was used as a positive control as different studies report spatial memory impairments following i.c.v. injection of scopolamine (Abbasi et al., 2013; Tabari et al., 2016). In line with literature, we observed impairments in cognitive function with scopolamine in both experimental protocols, implicating that the Y maze protocol is sensitive to impairments after i.c.v. delivery of compounds. We also demonstrated an increase in exploratory behavior following i.c.v. scopolamine injection, which complies with a previously published report stating that scopolamine can induce non-cognitive effects, such as increased locomotor activity and hyperactivity (Mathur et al., 1997). Nevertheless, it should be emphasized that the effect of scopolamine does not directly correlate with gliotransmission as scopolamine is a cholinergic M1 (muscarinic) receptor antagonist.

Next, we tested TAT-Gap19 in both set-ups. In the spontaneous alternation test, TAT-Gap19 did not lead to locomotor impairments or deficits in the spatial working memory compared to its inactive mutant version TAT-Gap19I130A. Likewise, Stehberg et al. (2012) observed no impairment in locomotor activity following TAT-L2 injection. In the delayed version of the Y maze, TAT-Gap19 significantly decreased the spatial short-term memory, since we observed a decrease in time spent in the novel arm of about 10%. This decline was in the same order of magnitude as the decline induced by scopolamine (~16%). On the contrary, Stehberg et al. (2012) found no impairment in short-term memory due to Cx43 HC inhibition by TAT-L2. However, this research group assessed short-term memory as an increase in freezing behavior in a fear memory paradigm which is mostly amygdala-dependent and not regulated by the dorsal hippocampus, unlike spatial memory.

By injecting biotin-TAT-Gap19, we observed peptide diffusion in the Cornu Ammonis 3 (CA3) region of the dorsal hippocampus. Distribution of biotin-TAT-Gap19 and TAT-Gap19 are presumed to be similar due to the small difference in molecular weight between these peptides (2929.61 vs. 2703.28 Da). As it is known that the dorsal hippocampus (e.g., CA3) is essential once the critical time window requires spatial memory for a longer time period (i.e., 5 min; Lee and Kesner, 2003; Yoon et al., 2008; Shipton et al., 2014), it is not surprising that TAT-Gap19 impairs spatial short-term memory. Indeed, as discussed further, hippocampal astrocytes mediate spatial memory through the release of gliotransmitters (Hassanpoor et al., 2014). TAT-Gap19 presumably does not affect spatial working memory since the hippocampus and medial prefrontal cortex jointly contribute to working memory/cognition (Moser et al., 2015) and other associational sensory cortices than the dorsal hippocampus could still provide information on sensory cues to the medial prefrontal cortex (Lee and Kesner, 2003). Furthermore, in the hippocampus, the structured network of place cells and grid cells (Moser et al., 2015), which is crucial for spatial memory formation, might be more vulnerable for gliotransmitter modulation (Hassanpoor et al., 2014).

A limitation of this study is that immunoreactivity for biotin-TAT-Gap19 could not be demonstrated exclusively in astrocytes. Several biotin-positive cells are apparently not GFAP-positive. However, while Cx43 is identified in various central nervous system cell types (i.e., developing neurons, activated microglia, pericytes and endothelial cells; Chew et al., 2010), the observed effect is most likely attributed to astrocytic Cx43 HCs, as Cx43 is predominantly and abundantly found in astrocytes and it is known that gliotransmission through astrocytes is pivotal for learning and (spatial) memory (Hassanpoor et al., 2014). Undoubtedly, astrocytes exert significant effects on synaptic activity and hippocampal neuronal output by ensheathing synapses of these neurons and by releasing gliotransmitters, which have diverse effects on adjacent neurons including place and grid cells (Hassanpoor et al., 2014). The contribution of Cx43 HCs on activated microglia and endothelial cells is most likely negligible, because Cx43 expression on these cell-types is correlated to brain injury and inflammation (Orellana et al., 2009). Our present experiments were however performed in healthy mice, despite the surgical procedure. Indeed, a recent study by Seyer et al. (2016) demonstrated that spatial working memory was unaffected 1 week after the implantation of an indwelling cannula into the lateral ventricle of rats. On day 6-post surgery, there was no difference in spontaneous alternation score, nor in number of arm entries, assessed in a plus maze. This finding was supported by the relatively small amount of neuronal cell death, limited spread of immune cell activation from the cannulation site and the lack of inflammatory gene upregulation in any area. The authors conclude that there is a very localized inflammatory response that does not extend beyond the immediate site of cannulation. The lack of cognitive deficit due to i.c.v. cannulation might be explained by the fact that the cannula does not descend deep into the brain. By this procedure, only the motor/somatosensory cortex is damaged and these areas are not specifically associated with memory (Seyer et al., 2016). Complementary, we could not find an influence of cannulation on spatial short-term memory as there was no difference in time spent in the novel arm between naïve NMRI mice (without i.c.v cannula) receiving an i.p. injection of 0.9% NaCl and i.c.v. cannulated NMRI mice receiving PBS i.c.v (on day 7-post surgery; data not shown). Additionally, studies demonstrating a clear role for acute modulation of endothelial cells or pericytes in spatial memory are still lacking. Therefore, the observed effect is most likely attributed to astrocytic Cx43 HCs.

In summary, our results complement previous studies and indicate for the first time that Cx43 HC inhibition impairs hippocampal short-term spatial memory. However, the results are still preliminary and further research is indicated to fully elucidate the regulatory pathway of Cx43 HCs. For instance, the results don’t necessarily mean that gliotransmitters are released directly through Cx43 HCs. Open HCs can facilitate Ca^2+^ entry (Schalper et al., 2010) that in turn could trigger Ca^2+^-dependent exocytosis of gliotransmitters indirectly (Martineau et al., 2014). Indeed, the exocytotic secretory machinery (e.g., soluble N-ethyl maleimide-sensitive fusion protein attachment protein receptor (SNARE) complex) is present in astrocytes (Hamilton, 2010) and astrocytic Ca^2+^-dependent exocytosis of glutamate, D-serine and ATP has been described in literature (Harada et al., 2016). Additionally, it cannot be excluded that alternative Ca^2+^-dependent release mechanisms are involved such as release through volume-regulated anion channels (Hamilton, 2010). Additionally, changes of intracellular Ca^2+^ can modulate other trafficking events in the constitutive secretion and recycling pathways and an increase in Ca^2+^ could release transmitters from astrocytes by promoting the insertion into the plasma membrane of proteins which mediate non-exocytotic release (Hamilton, 2010).

## Author Contributions

LW contributed to the design of the study and performed all the experiments. She acquired the data, performed the data analyses and interpreted the results. LW wrote the draft of the manuscript and created the artwork. MV contributed to the interpretation of the results. GA and DDB helped with optimizing the immunohistochemistry protocol. LL contributed to the conception of the study, the interpretation of data and the artwork. IJS supervised the project. She contributed to the conception and design of the study and the interpretation of the data. All authors revised the manuscript and gave final approval of the version to be submitted and any revised version. All authors agree to be accountable for all aspects of the work in ensuring that questions related to the accuracy of integrity of any part of the work are appropriately investigated and resolved.

## Conflict of Interest Statement

The authors declare that the research was conducted in the absence of any commercial or financial relationships that could be construed as a potential conflict of interest.
